# Supplementary material for: A New Cell Line from the Brain of Red Hybrid Tilapia (Oreochromis spp.) for Tilapia Lake Virus Propagation
Source: Animals (Basel). 2024 May 22;14(11):1522. doi: 10.3390/ani14111522 (PMC11171066; doi:10.3390/ani14111522)
Supplement: Supplementary file 1 [file animals-14-01522-s001.zip › Supplementary-Table-S1.pdf]

Supplementary Table S1. Genetic distance estimates among fish species using *cox1* nucleotide sequences

	OQ351327 RHTiB	MK335911 *C. gariepinus*	MK577976 *C. batrachus*	MZ871112 *S. asotus*	HM776365 *H. nobilis*	JX983319 *H. molitrix*	HM776360 *C. idella*	MT805055 *C. carpio*	MH515232 *O. niloticus*	MH515233 *O. niloticus*	MH515196 *O. niloticus*	MH515194 *O. niloticus*	MK497105 *O. shiranus*	MK497067 *O. mossambicus*	MK497065 *O. mossambicus*	MT418256 *O. mossambicus*	MT418255 *O. mossambicus*	MK497095 *O.mossambicus*
OQ351327 RHTiB																		
MK335911 *C. gariepinus*	0.3006339895																	
MK577976 *C. batrachus*	0.2790968209	0.1440147753																
MZ871112 *S. asotus*	0.2785500846	0.2341996506	0.2602606195															
HM776365 *H. nobilis*	0.2788490977	0.2874823254	0.2593055810	0.2926995451														
JX983319 *H. molitrix*	0.2768874096	0.2874823254	0.2625770243	0.2973799916	0.0016130908													
HM776360 *C. idella*	0.2568864186	0.2926965864	0.2627313305	0.2458730064	0.0988475650	0.1086040630												
MT805055 *C. carpio*	0.2814719018	0.2860611538	0.2572243232	0.2792644219	0.1765725905	0.1765725905	0.1518334219											
MH515232 *O. niloticus*	0.0093954333	0.3009915654	0.2933881995	0.2603542305	0.2659290565	0.2735376968	0.2435768654	0.2847411050										
MH515233 *O. niloticus*	0.0093954333	0.3009915654	0.2933881995	0.2603542305	0.2659290565	0.2735376968	0.2435768654	0.2847411050	0.0012552945									
MH515196 *O. niloticus*	0.0093954333	0.3009915654	0.2933881995	0.2603542305	0.2659290565	0.2735376968	0.2435768654	0.2847411050	0.0012544941	0.0012549698								
MH515194 *O. niloticus*	0.0062466622	0.3006339895	0.2879137646	0.2603542305	0.2656897385	0.2732760212	0.2410370611	0.2814719018	0.0037740507	0.0037754881	0.0037730718							
MK497105 *O.*	0.0872707841	0.2992008848	0.2590377582	0.2824283897	0.2354124080	0.2441063336	0.2440269823	0.3043141893	0.0734764562	0.0734764562	0.0734764562	0.0768128875						

shiranus																		
MK497067 *O. mossambicus*	0.0931155964	0.3120021148	0.2868911662	0.2728574063	0.2443009151	0.2558359468	0.2456017925	0.2868622451	0.0771449093	0.0771449093	0.0771449093	0.0805157794	0.0332908883					
MK497065 *O. mossambicus*	0.0913195219	0.3120021148	0.2868911662	0.2728574063	0.2443009151	0.2558359468	0.2456017925	0.2868622451	0.0754913579	0.0754913579	0.0754913579	0.0788541279	0.0317457952	0.0014655447				
MT418256 *O. mossambicus*	0.0967216630	0.3120021148	0.2918680343	0.2773360641	0.2572173212	0.2614860436	0.2512312090	0.2868622451	0.0840934934	0.0840934934	0.0840934934	0.0878674099	0.0334918828	0.0016198525	0.0000000000			
MT418255 *O. mossambicus*	0.0967216630	0.3120021148	0.2918680343	0.2773360641	0.2572173212	0.2614860436	0.2512312090	0.2868622451	0.0840934934	0.0840934934	0.0840934934	0.0878674099	0.0334918828	0.0016198525	0.0000000000	0.0000000000		
MK497095 *O. mossambicus*	0.0913195219	0.3120021148	0.2868911662	0.2728574063	0.2443009151	0.2558359468	0.2456017925	0.2868622451	0.0771449093	0.0771449093	0.0771449093	0.0805157794	0.0332908883	0.0029339264	0.0014655447	0.0000000000	0.0000000000	
