# Supplementary figures and images for: A New Cell Line from the Brain of Red Hybrid Tilapia (Oreochromis spp.) for Tilapia Lake Virus Propagation
Source: Animals (Basel). 2024 May 22;14(11):1522. doi: 10.3390/ani14111522 (PMC11171066; doi:10.3390/ani14111522)

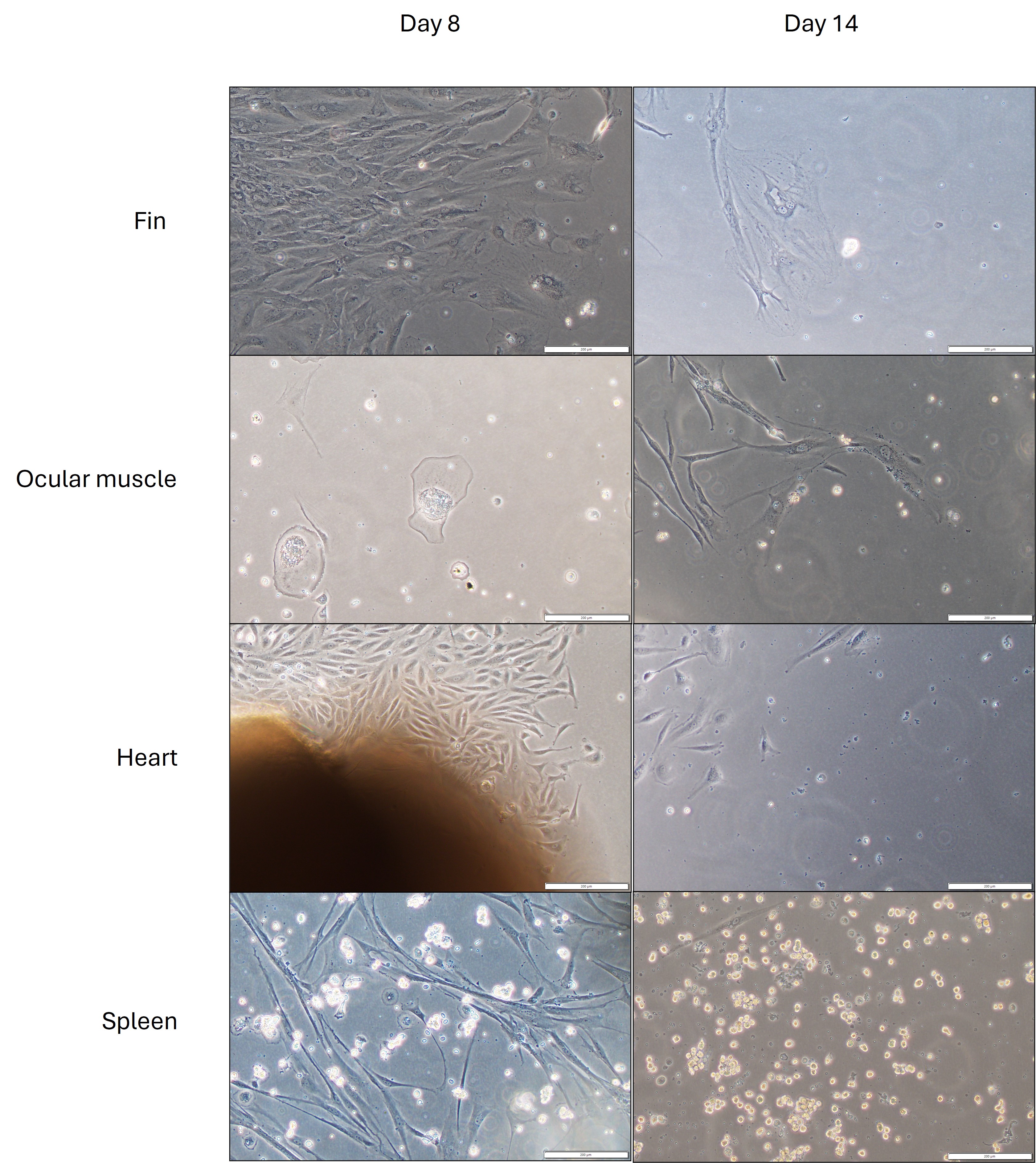

Supplement: Supplementary file 1 [file animals-14-01522-s001.zip › Supplementary Fig S1.jpg]

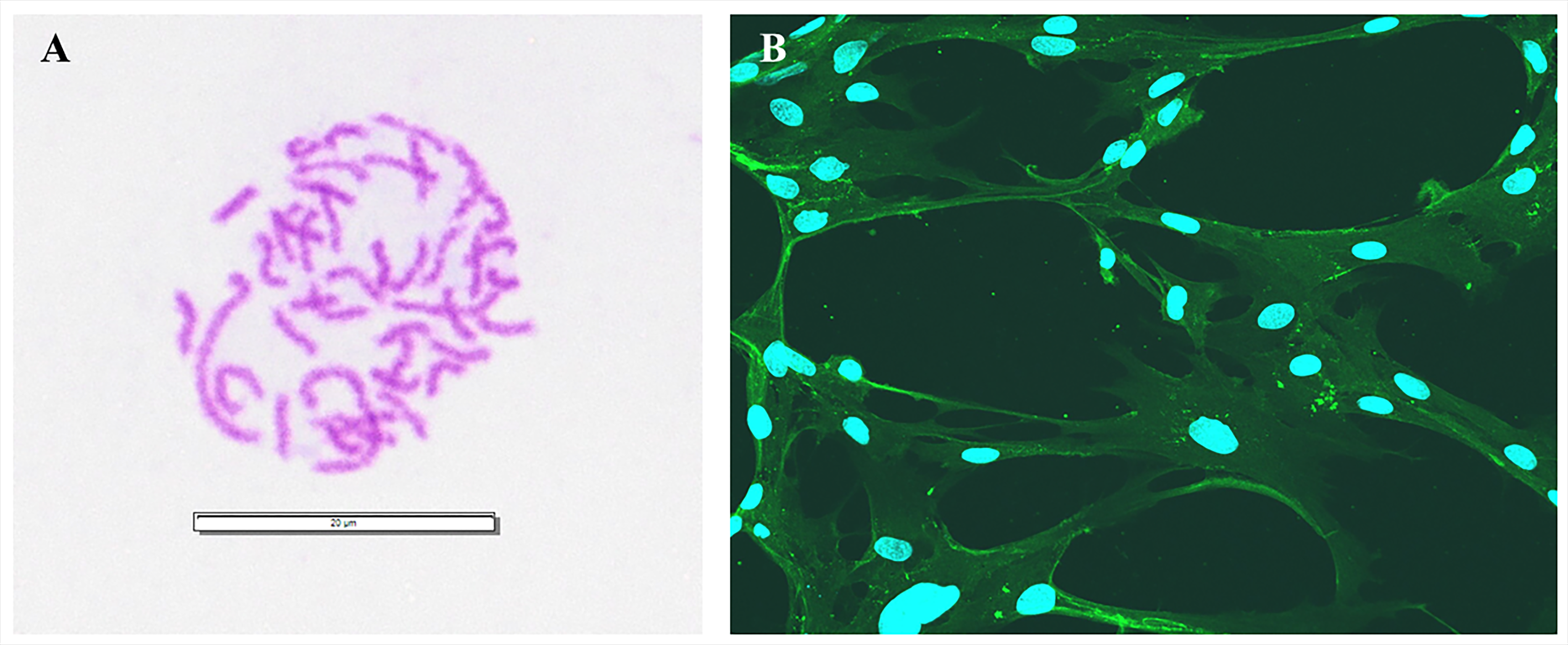

Supplement: Supplementary file 1 [file animals-14-01522-s001.zip › Supplementary Fig S2.tiff]
